# Physical activity and sedentary behavior impacts on dietary water intake and hydration status in Spanish schoolchildren: A cross-sectional study

**DOI:** 10.1371/journal.pone.0208748

**Published:** 2018-12-31

**Authors:** Aránzazu Perales-García, Rosa M. Ortega, Rafael Urrialde, Ana M. López-Sobaler

**Affiliations:** 1 Department of Nutrition and Food Science; Faculty of Pharmacy, Complutense University of Madrid, Madrid, Spain; 2 VALORNUT Research Group, Complutense University of Madrid, Madrid, Spain; 3 Health and Nutrition Department; Coca-Cola Iberia, Madrid, Spain; Nottingham Trent University, UNITED KINGDOM

## Abstract

**Background:**

The importance of maintaining an adequate hydration status and the complexity of the water balance make it necessary to study the lifestyle factors that can influence or modify these variables. The aim of this study was to evaluate the hydration status and dietary water intake for a sample of Spanish schoolchildren from 7 to 12 years old and their associations with physical activity (PA) and sedentary behaviors.

**Methods:**

A total of 242 schoolchildren was studied (49.17% females). A 24-hour urine sample was collected from each child, and the urine volume and osmolality were measured to estimate the hydration status (adequate hydration ≤800 mOsm/kg). In addition, a 3-day dietary record was completed to evaluate their water intake according to EFSA parameters. Dietary information was processed with DIAL software, and a statistical analysis was performed using SPSS. Student's t-test was used to study the normal variables, and the Mann-Whitney U test was used for those that were not normal. In the case of the categorical variables, the Z and Chi-Square proportions tests were used. The Bonferroni test was used to adjust the values in multiple comparisons. To evaluate the influence of these lifestyles on the urine osmolality, a 2-way ANOVA and an odds ratio were used.

**Results:**

A total of 48.3% of the sample presented an adequate hydration status, and the percentage was significantly higher in females (p = 0.003). Only 20.9% showed an adequate intake of dietary water. The lifestyle influence reveals that more inactive and non-sedentary schoolchildren were adequately hydrated (p = 0.008). PA (practice ≥1 h/day) was associated with a higher risk of having an inadequate hydration status, even when accounting for sex and other lifestyle factors (OR = 1.753(1.006–3.054), p = 0.048).

**Conclusions:**

Approximately half of the participants presented an inadequate hydration status (which was higher in males than in females). PA (practice ≥1 h/day) was associated with a higher risk of inadequate hydration. This fact highlighted the importance of raising awareness about hydration in children, especially in ones who are more active.

## Introduction

Water is practically essential for all of the functions of our bodies. Its regulation is based on a complex endocrine system that balances its intake (through food, beverages and metabolic water production) and loss (through skin, sweat, lungs, urine and feces) [1].

Maintaining an adequate hydration status is crucial because of water’s role in our bodies. The amount lost in urine depends on water consumed, the solute content of diet (high intakes of salt or protein will increase the daily fluid replacement because of the limited capacity of the kidneys to concentrate urine) and on the total losses [2]. If water intake is restricted, the kidneys will conserve water by producing more concentrated urine [3]. Equally, the body cannot store excess water, so the kidneys get rid of any temporary excess by producing a large volume of dilute urine [4].

Adequate hydration in schoolchildren can be related to better performance at concentration tasks [5] and better physical performance [4], especially in hot environments [6]. Healthy children may also be at risk of dehydration if there is a sudden increase in water loss for any reason, and physically active children will be at particular risk during periods of warm weather. The large surface area:volume ratio of children means they gain more heat through the skin when the environmental temperature exceeds skin temperature. In this situation, an increased rate of evaporative cooling is achieved at the expense of an increased loss of water from the body [4].

Currently, there is no “gold standard” method to determine an individual's hydration status, although it is important to the human body. The latest studies establish urinary osmolality and dietary water intake as two of the methods that are more frequently used [7]. Additionally, American College of Sports Medicine [8] also recommends thirst or urine color scales and plasma osmolality. In our study, thirst scales have not been considered appropriate because of the complexity of filled the questionnaire by parents through the impressions of children. Urine color scales have not been taking into consideration to evaluate the hydration status due to the lack of facilities in our study circumstances to realize an assessment of the urine sample by trained professionals on-site, who realized the anthropometry. Plasma osmolality is also an excelled standard, but it could be considered an expensive and invasive method [9] and urine osmolality has been considered a valid and reliable indicator of hydration status and could be used in field settings [10]. For this reason, urine analysis has been chosen as a practical, fast and less expensive method [11].

The dynamicity of the water balance makes it especially important to study factors that may modify it; physical activity (PA) and sedentary behaviors can be included among these factors [12, 13].

Regarding the role of inadequate hydration in PA, Stachenfeld et al. [14] highlighted the importance of improved thermoregulation through the maintenance of blood volume and the amount of sweat produced. In this sense, the sweat volume produced during PA practice directly affects the hydration status [15]. Despite the massive amount of knowledge about the physiological inter-regulation between these factors, very few studies have evaluated the hydration status in schoolchildren who maintain active lifestyles and perform PA for recreational purposes and in an unplanned manner [16]. Assessing physical activity behaviors in children is a complex issue. The nature of children’s physical activity behaviors (short, intermittent and, in some cases, unstructured) make it difficult to measure children’s PA [17]. The most feasible measures for schoolchildren are indirect reports by parents or teachers or objective methods such as direct observations, heart rate monitors or motion sensor such as accelerometers [18].

Sedentary leisure behaviors are gaining notoriety among the lifestyle factors. Currently, sedentary behaviors are considered an independent factor of PA that could be interacting with dietary and hydration patterns. Sedentary behavior is any waking behavior characterized by an energy expenditure ≤ 1.5 metabolic equivalents (METs), while in a sitting, reclining or lying posture [19]. Very few studies relate certain sedentary behaviors to different intake of food and beverages (in a quantitative or qualitative manner). This interaction could affect the hydration status. Therefore, we consider it especially interesting to study dietary habits, PA and sedentary behaviors in order to quantify the influence of lifestyle factors on hydration.

The aim of the present study was to evaluate the hydration status and dietary water intake in a sample of 7- to 12-year-old schoolchildren and their association with different lifestyles according to their PA and sedentary behaviors.

## Materials and methods

The design, recruitment of subjects, and methodology regarding anthropometry and urine analysis have been described elsewhere already [20]. The study protocol was approved by the Ethics Committee for Clinic Review of the Clinic San Carlos Hospital, which is part of the Complutense University of Madrid (Madrid, Spain) (Ref 15/522-E).

### Subjects

This observational study involved eight primary schools from various Spanish provinces (Madrid, Córdoba, Segovia and Ciudad Real), including the capital of the each province and a semi-urban/rural city. All schools were chosen randomly, looking to include children from different locations of the Spanish geography. Of the several schools contacted by telephone, eight accepted the invitation to be included in the study. Permission was requested to meet the parents of children in the age group 7–12 years. Once permission was given, the details of the study were explained to parents and all questions answered. Written informed consent was then sought to include their children in the study [20]. After this step, lifestyle information and a 3-day dietary record were collected, and the necessary material to collect 24-hour urine samples (24-h) was provided.

A total of 1,232 children was given the opportunity to participate in the study. Of these, 278 children provided written informed consent; 16 were excluded because they did not collect the urine sample correctly and 20 were excluded because they did not fully complete the lifestyle questionnaire. The final sample, therefore, compromised 242 from 7 to 12 years old; 49.17% of them were females, and 50.83% were males. The fieldwork was performed between February and March 2014, when the average temperatures were 13.5°C and 14.5°C, respectively and the range of temperatures were 3.6–14.7°C in February and 4.6–16.8°C in March.

The inclusion criteria were as follows: living in the provinces of the study, being between 7 and 12 years old and attending the second to sixth grades of primary school, being in compliance with the informed consent via parents or legal guardians, and not presenting any clinical problem that could modify the results, such as liver or kidney diseases, diabetes, and/or hyper/hypokalemia. In addition, the participants had received no pharmacological treatments in the 3 months prior to the study with corticosteroids, insulin or diuretics.

### Anthropometric data

All measurements took place at the schools in the mornings and in accordance with norms set out by the WHO (World Health Organization) [21]. The weights and heights were measured using a digital electronic balance (range 0.1–150 kg; precision, 100 g; Alpha; Seca, Igni, France) and a digital stadiometer (70–205 cm; 1mm; Harpenden Pfifter, Carlstadt, NJ, USA). The body mass index (BMI) was then calculated and compared with the IOTF cut-off points [22].

### Lifestyle study

A lifestyle questionnaire [23], previously applied in other Spanish populations of children and adults [24, 25, 26], was filled out by the parents or legal guardians (S1 Appendix). The questionnaire included questions to quantify the primary activities that could indicate an active lifestyle and sedentary behaviors, both on weekdays and weekends. This questionnaire collected the frequency of active play, physical activity as a subject in school, extracurricular physical activities and the time spent on each activity. Additionally, questions about the hours spent in front of different displays were used as an indicator of sedentary behavior (personal computer/video game console/television), both on workdays and weekends. Parents, legal guardians or caregivers of schoolchildren collected this information through active observation except for physical activity as subject in school and extracurricular physical activities. In the last two cases teachers provided the information. In the case of active plays, information provided by schoolchildren on the activities carried out during the break has also been taken into account. When all of the information was collected, the reported mean of PA hours per week was calculated (activities that increase the energy expenditure above the BMR (Basal Metabolic Rate) according to the Compendium of Physical Activities (2011) [27]. These activities included: active play, PA performed in school and extracurricular physical activities) and sedentary behaviors (hours spent in front of the PC (personal computer), video game console and TV (television)).

To estimate the time spent on PA, a classification of children into “actives” was created for those participating in greater than the WHO's recommendation of 1 hour or more of performing moderate to vigorous physical activities to maintain health from 5 to 17 years [28]. However, children were classified as “sedentary” when they spent more than 2 hours performing this type of sedentary activities, according to the latest guidelines of the Australian government for children within the same age range [29]. Afterwards, a classification of the study sample was performed by accounting for these two variables to establish the four following different groups: “sedentary active”, “non-sedentary active”, “sedentary inactive” and “non-sedentary inactive”.

### Dietetic study

Parents or legal guardians filled out a dietary record [30] about the consumption of food and beverages by the schoolchildren over 3 days (2 weekdays and 1 weekend day). The days chosen to collect the information were Thursday and Friday as weekdays and Sunday and weekend day. The questionnaire collected the amounts of food and beverages consumed and the time of each intake. In the case of lunch on weekdays that was not consumed with parents, the information collected was checked against the menus provided by school catering service. Once collected, the data were processed in DIAL software for nutritional assessment [31]. The results were focused on estimating the dietary water intake (coming from food and beverages, expressed in ml/day), the percentage of water coming from beverages, and the ml/day of different beverages consumed, from water as a beverage, sugary drinks, sweetened drinks, sports drinks, natural juices, commercial juices and nectars, milk and milkshakes. Specifically, water as a beverage is defined as water in the form of tap water, mineral water and sparkling mineral water. The dietary water intake classification was established on the basis of the Adequate Intake (AI) by the European Food Safety Authority (EFSA) [32], which is 1,600 ml/day for 4-8-year-old children, 1,900 ml/day for females 9 to 13 and 2,100 ml/day for males aged 9 to 13.

### Urine testing

A 24-hour urine sample was collected on a weekend day, and it coincided with the dietary record. All of the participants received written instructions and individual containers for the correct collection of the sample. All micturitions were stored immediately in preservative-free, 1-L plastic containers at temperatures <-12°C before transfer to the laboratory. The volume as well as the urinary urea [33], sodium, potassium [34] and creatinine [35] were determined. To confirm appropriate collection of 24-h urine, the correlation between urinary levels of creatinine and muscular mass of each subject was taken into account. Fat-free mass was calculated bearing in mind the creatinine excreted over 24 h in urine using the following equation [36]:

Fat‑freemass(kg)=0.02908Xcreatinine(mg/day)+7.38

The osmolality was calculated according to the following formula: Osmolality = [{sodium (mEq/l) + potassium (mEq/l)}*2 + urea (mg/dl)]/5.6 [37]. The children were considered to have adequate hydration (AH) when the osmolality was equal to or lower than 800 mOsm/kg in accordance with the criteria of other authors [38, 39, 40, 41, 42].

### Statistical analysis

The data were analyzed using the statistical software IBM SPSS Inc. (version 21.0). Descriptive analysis of the values shows the mean ± standard deviation (m±sd). The Kolmogorov-Smirnov test was used to test the normality of the variables. Student's t-test was used to study the normal variables, and the Mann-Whitney U test was used for those that were not normal. For the categorical variables, the Z and Chi-Square proportion tests were used. The Bonferroni test was used to adjust the values in multiple comparisons. To evaluate the influence of the lifestyles, a 2-way ANOVA was performed on the urinary osmolality and different biochemical parameters, and the water dietary intake and beverages were explored with a post hoc Bonferroni analysis. The association of an individual’s sex, compliance with the practice of PA and sedentariness (independent variables) and risk of dehydration (dependent variable) were analyzed using a logistic regression analysis to calculate three models of the odds ratio (OR). In addition, the OR adjusted by sex and the OR adjusted by sex and other lifestyle factors were calculated. Statistical differences were assigned for a value of p <0.05.

## Results

The sample description is shown in Table 1. The males performed significantly more hours of total physical activities and extracurricular activities, in addition to a greater number of hours of PC/video game console/TV use than females. To this finding, we must add that the percentage of males who complied with the PA guidelines is higher than the percentage for females, without significant differences in compliance with the sedentary guidelines.

**Table 1:** Descriptive table of the sample.

	Total (n = 242)	Female (n = 119)	Male (n = 123)	p^a^ value
Age (years) (†)	8.9±1.2	8.9±1.2	8.9±1.1	0.853
**Anthropometric Data**				
Weight (kg)	36.0±9.0	36.0±8.0	36.0±9.0	0.667
Height (cm)	137.6±8.9	137.1±9.7	138.1±8.0	0.421
BMI (kg/m2) (†)	18.7±3.2	18.7±2.9	18.7±3.5	0.498
Weight situation (IOTF) (%)				0.082
Under-weight	2.9	4.2	1.6	
Normal-weight	59.1	55.5	62.6	
Overweight	27.3	32.8	22.0	
Obese	10.7	7.6	13.8	
**Physical activity indicators**				
**Total activity (hours/day)**	2.44±1.14	2.20±1.11	2.66±1.13	**0.002**
Active play (hours/day)	1.53±0.97	1.39±0.93	1.67±0.99	**0.024**
Physical activity as a subject in school (hours/day) (†)	0.55±0.41	0.53±0.43	0.58±0.39	0.089
Extracurricular physical activity (hours/day) (†)	0.44±0.37	0.38±0.34	0.50±0.39	**0.006**
**Adherence to PA guidelines**				
Practice ≥ 1 hour of PA/day (%)	65.3	56.3	74.0	**0.004**
**Sedentary indicators**				
**Sedentary time (hours/day)**	3.86±1.50	3.72±1.25	4.00±1.69	0.148
Use of PC/console (†)	1.44±0.96	1.25±0.77	1.63±1.09	**0.012**
Use of TV (†)	2.42±0.94	2.47±0.94	2.37±0.94	0.268
**Adherence to sedentariness guidelines**				
≤2 hours of sedentariness/day (%)	11.2	11.8	10.6	0.786

m: mean; sd: standard deviation; PA: Physical activity; BMI: Body Mass Index. All data are shown as m±sd unless otherwise stated.

(a) Significant differences according to sex (p <0.05) found by Mann-Whitney (†), Student's t-tests or by the Chi-Square test and the Z-test of proportions if they are percentages.

Table 2 shows a description of the different parameters related to hydration and the dietary water intake studied here. The results showed that male schoolchildren had a greater excretion of creatinine, urea, sodium and potassium, in addition to higher urinary osmolality.

**Table 2:** Descriptive table of hydration data and dietary water intake of the sample.

	Total (n = 242)	Female (n = 119)	Male (n = 123)	p^a^ value
**Hydration data**				
**Biomarkers**				
Diuresis (ml/24-h)	882.0±290.0	877.5±305.4	887.2±276.7	0.780
Creatinine (mg/24-h)	720.2±203.7	677.1±206.1	761±192	**0.001**
Urea (g/24-h)	17.5±5.5	16.2±5.1	18.8±5.6	**0.000**
Sodium (mEq/24-h)	133.5±52.3	124.5±45.3	141±57	**0.011**
Potassium (mEq/24-h)	49.0±15.9	47.1±15	52±17	**0.012**
Osmolality (mOsm/kg)	804.7±205.3	764.3±204.4	843.7±199.4	**0.002**
Adequate hydration status (≤ 800 mOsm/kg) (%)	48.3	58.0	39.9	**0.003**
**Dietary water intake data**				
Total dietary water intake (ml/day)	1,412±431	1,357±377.7	1,465±473.1	0.051
Water from beverages (%) (†)	58.7±17.9	57.6±16.6	59.7±19.2	0.529
Adequate water intake compliance (%)	20.9	17.9	23.8	0.269
**Types of beverages (ml/day)**				
Water as a beverage^b^ (†)	431.3±366.3	414.9±337.5	447.2±392.8	0.979
Sugary drinks (†)	36.9±68.0	37.87±71.6	36.07±64.6	0.783
Sweetened drinks (†)	13.1±45.7	8.5±31.4	17.59±55.9	0.209
Sports drinks (†)	15.7±50.7	16.6±58.8	14.75±41.6	0.891
Natural juices (†)	39.8±68.4	36.6±66.0	42.89±70.8	0.616
Commercial juices and nectars (†)	77.0±99.2	67.1±88.8	86.6±107.8	0.147
Milk	282.5±139.1	273.7±135.2	294.2±142.2	0.099
Liquid yogurt and other fermented milks (†)	72.3±61.3	70.7±64.8	73.9±57.9	0.574
Milkshakes (†)	24.3±48.4	21.2±49.1	27.3±47.6	0.133

m: mean; sd: standard deviation, All data are shown as m±sd unless otherwise stated.

(a) Significant differences according to sex (p <0.05) found by the Mann-Whitney (†) or Student's t-test or Chi-Square test and the Z-test of proportions if they are percentages

(b) Water as a beverage is the combination of the following subgroups: tap water, natural mineral water and sparkling water.

Children with an inadequate hydration status (≤ 800 mOsm/kg) (IH) had higher sodium excretion (145,8±53,4 mEq vs. 119,4±46,2 mEq/24h vs. in AH, p<0.001), higher urea excretion (19,1±5,7 g/24h vs 15,9±4,7 g/24h in AH, p<0.001), while potassium excretion was similar. IH children had also lower diuresis (760,4±232,7 ml/24h vs. 1012,6±290,3 ml/24h in AH, p<0.001), while dietary water intake was similar in both groups. We performed a linear regression analysis to analyze the risk of dehydration considering all these variables. A higher diuresis was a protective factor of dehydration (OR = 0.948 (95%CI = 0.925–0.972)), while urea excretion (OR = 4.174 (2.096–8.315)) and sodium excretion (OR = 1.164 (1.078–1.257)) were risk factors of dehydration. On the other hand, water intake was not associated to the risk of dehydration.

Table 3 shows the biochemical and dietary results according to four lifestyle groups. With regard to the biochemical data, “non-sedentary inactive” schoolchildren presented a significantly higher percentage of adequate hydration. In the sedentary groups, creatinine and sodium excretion was higher. Notably, greater osmolality appeared in those who were more active.

**Table 3:** Hydration and water intake data according to the lifestyle classification.

	Inactive		Active		p^a^ value	Two-way ANOVA		
						PA	Sedentariness	Both
	Sedentary (n = 74)	Non-sedentary (n = 10)	Sedentary (n = 141)	Non-sedentary (n = 17)				
%	30.6	4.1	58.3	7.0				
Age (years)	9±1,2	9,3±1,2	8,9±1,1	8,6±1,3				
Males (%)	60.8	70.0	42.6	42.2				
**Hydration data**								
**Biomarkers**								
Diuresis (ml/24-h)	922±320	918±229	869±286	796±202		0.157	0.524	0.574
Creatinine (mg/24-h)	721±201	605±143	740±203	616±201		0.714	**0.005**	0.927
Urea (g/24-h)	17.1±4.9	15.7±5.3	18±5.8	16.3±5.4		0.525	0.184	0.893
Sodium (mEq/24-h)	132±51^c,d^	90.0±40.0^c^	139±53.0^d^	111±36.0^c,d^		0.200	**0.001**	0.509
Potassium (mEq/24-h)	50±15	52±18	50±17	46±12		0.454	0.829	0.402
Osmolality (mOsm/kg)	775±219^c,d^	622±168^c^	835±191^d^	791±217^c,d^		**0.008**	**0.021**	0.206
AH status (≤ 800 mOsm/kg) (%)	55.4	90.0	42.6	41.2	**0.014**			
**Dietary water intake data**								
Total dietary water intake (ml/day)	1,316±389.4	1,513±443	1,451±449	1,447±412				
Water from beverages (%)	53.9±15.1	57.0±20.1	60.3±17.8	66.5±25.2				
Adequate water intake compliance (%)	16.2	40.0	20.9	31.3	0.238			
**Types of beverages (ml/day)**								
Water as a beverage^b^	337±330	513±431	473±380	450±312		0.636	0.321	0.197
Sugary drinks	33.8±66.5	14.3±25.0	42.5±73.3	17.3±33.9		0.685	0.122	0.840
Sweetened drinks	13.3±47.0	0±0	14.8±48.5	5.9±24.3		0.704	0.253	0.821
Sports drinks	8.5±26.3	6.67±21.09	19.3±60.3	22.2±54.9		0.222	0.960	0.829
Natural juices	38.2±63.5	76.6±77.1	37.7±70.2	42.8±68.9		0.237	0.133	0.251
Commercial juices and nectars	73.0±95.3	75.0±92.0	79.7±103.4	73.2±90.9		0.910	0.912	0.839
Milk	307±150	201±180	270±130	329±110		0.121	0.422	**0.005**
Liquid yogurt and other fermented milks	67.8±63.0	85.7±52.4	77.4±61.6	42.3±48.0		0.192	0.506	**0.041**
Milkshakes	13.1±34.3	20.0±45.0	27.7±53.3	47.0±51.4		**0.041**	0.197	0.542

m: mean; sd: standard deviation; AH: Adequate Hydration- All data are shown as m±sd unless otherwise stated.

(a) Significant differences from the Chi-Square test.

(b) Water as a beverage is the combination of the following subgroups: tap water, natural mineral water and sparkling water.

(c), (d) Different superscript letters indicate statistical differences between groups.

Table 4 shows crude and adjusted ORs that relate different aspects of lifestyle to the risk of dehydration. After adjusting the OR, the only variable that maintained its association with a greater risk of dehydration (OR = 1.756 (1.008–3.060), p = 0.047) was PA (when performing 1 hour or more a day). However, the results showed that neither complying with the PA recommendation nor with the sedentary recommendation was related to non-compliance of the AIs (PA: sex-adjusted OR = 1.118 (0.568–2.000), p = 0.784 and sedentariness: sex-adjusted OR = 2.242 (0.930–5.406), p = 0.072).

**Table 4:** Association of sex, lifestyles and compliance with AI water guidelines with the prevalence of IH.

	Model 1	p value	Model 2	p value	Model 3	p value
	Odds Ratio (95% CI)		Odds Ratio (95% CI)		Odds Ratio (95% CI)	
**Sex **						
**Female**	1					
**Male**	2.156 (1.290–3.604)	**0.003**				
**PA compliance**						
**PA (< 1 hour/day)**	1		1		1	
**PA (≥ 1 hour/day)**	1.997 (1.166–3.420)	**0.012**	1.779 (1.026–3.086)	**0.040**	1.753 (1.006–3.054)	**0.048**
**Sedentary compliance**						
**Sedentariness (> 2 hours/day)**	1		1		1	
**Sedentariness (≤ 2 hours/day)**	0.609 (0.270–1.373)	0.232	0.612 (0.268–1.401)	0.246	0.541 (0.228–1.284)	0.163
**AI compliance**						
**Under AI (No)**	1		1		1	
**Above AI (Yes)**	1.137 (0.609–2.125)	0.686	1.067 (0.564–2.019)	0.842	1.114 (0.579–2.144)	0.746

PA: Physical activity; AI: Adequate intake; IH: Inadequate Hydration

(a) Model 1: Unadjusted Odds Ratio; Model 2: Odds Ratio adjusted by sex; Model 3: Odds Ratio adjusted by al variables.

## Discussion

The 24-h urine osmolality result was similar to that in other studies, in schoolchildren, as performed using the same methodology in different countries such as France [38], Egypt [39, 40], Greece [41], Portugal [42] and the United States [43].

We found that males had higher osmolality values than females. This result has been supported by several studies such as Ebner & Manz [44] in German schoolchildren (4–14 years old) from the DONALD study, Kavouras et al. [41] (Greece schoolchildren from 9–13 years old), Bonnet et al. [38] (French schoolchildren, 9–11 years old) and in Portuguese schoolchildren by Rodríguez et al. [42] (9–10 years old) and Padrao et al. [41] (7–11 years old). There are some factors that could explain these disparities between males and females. First, males show higher intake of food and energy, while females show higher intake of water-dense food, specifically fruits and vegetables [45, 46, 47]. Females also have a lower protein [48], sodium, potassium and phosphorus [49] intake and, consequently, lower total consumption of solutes. Additionally, the different PA patterns between the sexes could play a significant role on these disparities; before and after puberty, males also show a higher PA and higher non-renal water losses [44, 50].

Our results show that the percentage of schoolchildren who presented an osmolality ≤ 800 mOsm/kg was similar to the findings of other studies that employed the same cut-off point as Stookey et al. [43] (37% females and 34% males from Los Angeles and New York, from 9 to 11 years old) and Gouda et al. [40] (43% total, 44.8% females and 41.8% males from Egypt, from 9 to 11 years old).

Regarding the dietary data, the mean dietary water intake showed similar results to those of the ANIBES study [51] on Spanish schoolchildren from 9 to 12 years old, the Cuenca Study (Spanish schoolchildren from 9 to 11 years old) [52] and other international studies in Mexico [53] (5–11 years old), Canada [54] (4–8 and 9–13 years old) and France [55] (6–11 years old). Finally, our results were lower than those of Kavouras et al. [41] in Greek schoolchildren, when using 2-day beverage records to collect the quantity, the type and the timing of consumption for water and different beverages that could influence the disparity between the results. Sodium intake can also influence hydration status since the excretion of sodium excess requires the excretion of water. The best method to assess sodium intake is to measure the excretion of sodium in urine for 24 hours. The sodium intake in our population is higher than that recommended [20] and it was even higher in IH children. Urea excretion is also higher in IH, suggesting a higher protein intake. And finally, although water intake was similar in both IH and AH children, the former had also lower diuresis, which suggest that IH children could have higher extra-renal losses of water.

In evaluating the adequacy for the AI of the EFSA, our results showed lower percentages than those reported by Kavouras et al. [41], which included 48% of schoolchildren above the AI (50% females and 47% males). This difference could be based on the disparity between methodologies used to collect dietary water intake information.

### Physical activity

In accounting for the PA performed by the schoolchildren, more than 60% met the recommendations for total PAs. This percentage falls within similar ranges to those obtained in the ANIBES Study [56] in the 9- to 12-year-old cohort (51.6%; of those, 61.1% were males and 37.9% were females). Males had higher adherence to the recommendations, spending more time performing active play and extracurricular physical activities. Other authors also found statistical differences in these types of PAs according to sex, although comparisons between studies in no easy become there may be cultural aspects that affect boys’ and girls’ PA differently. Ishii et al. [57] studied a sample of Japanese schoolchildren; they observed the same differences in the type of PAs performed and the leisure physical activities based on sex, which were lower in females than in males. Yamamoto-Kimura et al. [58] studied Mexican schoolchildren; their results showed that males practiced more extracurricular physical activities than females.

Other relevant aspects of the results associate compliance with the PA recommendation and being a boy with a higher risk of IH in the study sample. Males may have a higher risk because of their body composition, which can present differences even at these ages, with more body fat and less body water than females [59, 60].

However, adherence to PA guidelines may be a risk factor for IH if the water intake does not increase proportionally to the losses associated with higher PA (in the case of our study, there are no differences in the water intake depending on the lifestyle). Additionally, other socioeconomic factors such as accessibility to beverages may also be influence on the hydration status, so they have to be considered as other potential risks of IH.

As far as we know, the effects of compliance with the PA guidelines on the state of hydration in schoolchildren have not been evaluated. Similar data have been found in the PHYSMED study [61] conducted in a Spanish adult population aged 55 to 88 years old in which, similar to the results obtained in the present study, significant differences were observed in the plasma osmolality between the "sedentary inactive" and "non-sedentary active" groups.

These data highlight the importance of raising awareness about the hydration status in schoolchildren and their environment in which the primary source of hydration should be water as a beverage. It is especially important when it comes to implementing a strategy to promote the performance of PA, with nutritional education playing a key role. Cleary et al. [62] showed a clear example of this promotion; they evaluated a sample of female adolescents who played volleyball as an extracurricular sports activity. These girls had IH on average before and after sports practice. This situation reversed over the period of time (4 weeks) during which they were subjected to nutritional education in the field of hydration for sports. During this training period, the results showed a suitable improvement in the individual hydration state after PA practice due to correct fluid replacement. Unfortunately, once the training was completed, the adolescents again had IH levels before and after sports practice. Kavouras et al. [63] designed an intervention program to increase fluid intake in children who exercise with heat. In this study the children had free access to fluids and water accessibility was facilitated in training, dinning and resting areas. During the intervention period hydration status and performance in an endurance run was improved. In both cases, the fluid replacement regime was developed by the health professionals who were conducting the study due to the lack of standardized hydration guidelines adapted to different sports. In this sense, it is vitally important to bear in mind that post-exercise hydration recommendations may vary depending on the sport and intensity. These specificities are not included in the current hydration guidelines. This finding suggests that fundamental support for this group, in addition to training, should be a commitment by professionals involved in sports, health professionals and the family environment. On the other hand, it is interesting to highlight the need to implement other strategists as promoting water consumption in schools in order to encourage AH in this group [64, 65].

### Sedentary behaviors

Our results indicate that sedentary behavior, unlike PA, did not increase the risk of IH, nor did it increase the risk of non-compliance with adequate water intake.

The lifestyle of the studied population included a low percentage of schoolchildren who complied with the sedentary guidelines. These results are consistent with those obtained by Rodríguez-Huertas et al. [66] in Spanish schoolchildren aged 6 to 12 years in which only 30% performed less than 2 hours a day of sedentary leisure activities and data from the ALADINO study [67], which showed that 37.3% of the population dedicates less than 1 hour, 33.3% dedicates approximately 1 hour, and 21.7% dedicates 2 or more hours to these sedentary activities. In addition, our results show that males commit significantly more of their time to PCs/video game consoles/TVs compared to females. De Jong et al. [68] have obtained similar results in their evaluation of the television and computer use habits of two school-aged population groups, from 4 to 8 years and from 9 to 13 years. Their results showed that computer use by this population increased as the age increased and with the presence of computers in the bedroom for males in both age groups.

Regarding the relationship between sedentariness and beverage consumption, it is very difficult to compare with other studies since some of them studied different sedentary behaviors and their relations to the consumption of drinks, but they do not show results for guideline compliance in general. The systematic review by Pearson et al. [69] concluded that television viewing was positively associated with the consumption of energy-dense beverages among other dietary patterns, both in schoolchildren and adolescents. In our country, the ALADINO Study [70] showed that more than 2 hours of screen time per day had a significant association with the increased frequency of consumption of smoothies, fresh fruit and sugary drinks.

It is of special relevance to note that these results may not be applicable to other subjects or population groups, but they provide an interesting line of research to evaluate the influence of lifestyle factors on hydration. Among the strengths of our study, we highlight the size of the study sample, which is a healthy school population, not athletes, and the collection of 24-h urine to determine the hydration status, which makes it possible to quantify both the diuresis in addition to the osmolality. However, self-reported data on PA and sedentary lifestyles are subject to error, as is the 3-day dietary record, and thus, there may have been problems in terms of the over/underestimation of some of the aspects studied here. In addition, the cross-sectional design of the study does not allow us to establish a cause-effect relationship between variables.

## Conclusions

Roughly half of the individuals studied here presented an IH status, and this percentage was higher in males than females. It is important to encourage schoolchildren to be active, and it also essential to reinforce the importance of hydration to these groups, taking into special account the awareness of the people in their environment in helping them to achieve this goal and in acquiring hydration habits to help them maintain an adequate hydration status.

## Supporting information

S1 Appendix Lifestyle questionnaire. Spanish and English versions.
(PDF)
