# Supplementary material for: Physical activity and sedentary behavior impacts on dietary water intake and hydration status in Spanish schoolchildren: A cross-sectional study
Source: PLoS One. 2018 Dec 31;13(12):e0208748. doi: 10.1371/journal.pone.0208748 (PMC6312295; doi:10.1371/journal.pone.0208748)
Supplement: S1 Appendix — Spanish and English versions. (PDF) [file pone.0208748.s001.pdf]

S1 Appendix. Lifestyle questionnaire. Spanish and English versions

UNIVERSIDAD COMPLUTENSE DE MADRID
DEPARTAMENTO DE NUTRICION

Facultad de Farmacia
Ciudad Universitaria
28040 Madrid
Tel: +34 91 394 18 10
Fax: +34 91 394 17 32

CUESTIONARIO DE ESTILO DE VIDA

(a rellenar por los progenitores/madres/tutores)

Nombre y apellidos del niño/a: ____

Persona que rellena el cuestionario: Madre ☐ Padre ☐ Otro (especificar): ☐ ....

Indique el tiempo (horas o minutos) empleado en la realización de cada actividad de forma que el tiempo total de cada una de las columnas sume 24 horas.

**Actividad**	**Día laborable (promedio de 5 días)**	**Fines de semana (promedio de 2 días)**
Dormir (incluir siestas)		
Actividades que se realizan sentado (estudiar, hablar con amigos, etc.)		
Comer (incluir todas las comidas realizadas en el día)		
Actividades que se realizan de pie (conversar, esperar, etc.)		
Pasear, andar, desplazamientos		
Jugar de forma activa (correr, patines, bicicleta, saltar, bailar, fútbol, etc.)		
Educación física realizada en el colegio		
Clases de deporte extraescolares (fútbol, tenis, baloncesto, equitación, baile, etc.)		
Ocio (ver televisión, jugar a videojuegos, jugar con el ordenador, iPad, móvil, whatsApp, etc.)		
Otros (especificar):		

¿A qué hora se levanta y se acuesta habitualmente su niño/a?

	**Días laborables**	**Fines de semana**
Hora a la que se levanta:	|___|___| Horas<br>|___|___| Minutos	|___|___| Horas<br>|___|___| Minutos
Hora a la que se acuesta:	|___|___| Horas<br>|___|___| Minutos	|___|___| Horas<br>|___|___| Minutos

En una semana normal, ¿cuántos días va su niño/a a actividades extraescolares de deporte (fútbol, tenis, baloncesto, baile, etc.)?:

Menos de un día a la semana		4 días a la semana	
1 día a la semana		5 días a la semana	
2 días a la semana		6 días a la semana	
3 días a la semana		7 días a las semana	

Indique el tiempo empleado a cada actividad por sesión:

**Actividades extraescolares de deporte (especificar)**	**Tiempo empleado por sesión**
	|___|___| Horas<br>|___|___| Minutos
	|___|___| Horas<br>|___|___| Minutos
	|___|___| Horas<br>|___|___| Minutos
	|___|___| Horas<br>|___|___| Minutos
	|___|___| Horas<br>|___|___| Minutos

Indique cuál/cuáles de estos dispositivos tiene en casa y está disponibles para su uso libre por su niño/a:

Ordenador	
Videoconsola	
Teléfono/móvil con Internet	
Televisión en la habitación del niño/a	

Indique cuantas horas dedica normalmente su niño/a a jugar con el ordenador, videoconsolas, o en internet en su tiempo libre, tanto en casa o fuera

**Días laborables**		**Fines de semana**	
Nada		Nada	
Menos de 1 hora por día		Menos de 1 hora por día	
Sobre 1 hora por día		Sobre 1 hora por día	
Sobre 2 horas por día		Sobre 2 horas por día	
Sobre 3 horas por día		Sobre 3 horas por día	
Sobre 4 horas por día		Sobre 4 horas por día	
Sobre 5 horas por día		Sobre 5 horas por día	
6 o más horas al día		6 o más horas al día	

Indique cuantas horas por día dedica habitualmente su niño/a a ver televisión, (incluyendo videos) en su tiempo libre, en casa o fuera:

**Días laborables**		**Fines de semana**	
Nada		Nada	
Menos de 1 hora por día		Menos de 1 hora por día	
Sobre 1 hora por día		Sobre 1 hora por día	
Sobre 2 horas por día		Sobre 2 horas por día	
Sobre 3 horas por día		Sobre 3 horas por día	
Sobre 4 horas por día		Sobre 4 horas por día	
Sobre 5 horas por día		Sobre 5 horas por día	

(ENGLISH VERSION)

LIFESTYLE QUESTIONNAIRE

(to be completed by parents/guardians)

Name and surname of the child: ____

What is your relationship to the child?: Mother ☐ Father ☐ Other (specificy): ☐ ....

Indicate the time (hours or minutes) spent performing each activity so that the total time of each of the columns totals 24 hours.

Activity	Weekdays (Average of 5 days)	Weekends (Average of 2 days)
Sleep (including naps)		
Activities that are done sitting (studying, talking to friends, etc.)		
Eat (include all meals taken during the day)		
Activities that are carried out standing (talking, waiting, etc.)		
Go for a walk, move from one place to another walking		
Playing actively (running, skating, cycling, jumping, dancing, soccer, etc.)		
Physical education carried out in the school		
Extracurricular sports classes (football, tennis, basketball, horse riding, dancing, etc.)		
Leisure (watching TV, playing video games, playing computer games, iPad, mobile phone, whatsApp, etc.)		
Other (please specify):		

What time does your child usually get up and go to bed?

	Weedays	Weekend
Time at which the children gets up:	|___|___| Hours<br>|___|___| Minutes	|___|___| Hours<br>|___|___| Minutes
Time at which the children go to the bed:	|___|___| Hours<br>|___|___| Minutes	|___|___| Hours<br>|___|___| Minutes

Over a typical or usual week, on how many days does your child go to this/these sport or dancing club(s)? (soccer, tennis, basketball, dancing, etc.)?

Less than one day a week		4 day per week	
1 day per week		5 day per week	
2 day per week		6 day per week	
3 day per week		7 day per week	

Indicate the time spent on each activity per session:

Extracurricular sports activities (specify)	Time spent per session
	|___|___| Hours<br>|___|___| Minutes
	|___|___| Hours<br>|___|___| Minutes
	|___|___| Hours<br>|___|___| Minutes
	|___|___| Hours<br>|___|___| Minutes
	|___|___| Hours<br>|___|___| Minutes

Indicate what devices are available to your child at home:

Computer	
Videoconsole	
Telephone with internet	
Television in the child's room	

How many hours a day does your child usually spend using a computer, video game consoles, or other devices, for playing games, , whether at home or elsewhere, in his/her free time?

Weekdays		Weekends	
Never		Never	
Less than 1 hour per day		Less than 1 hour per day	
About 1 hour a day		About 1 hour a day	
About 2 hours a day		About 2 hours a day	
About 3 hours a day		About 3 hours a day	
About 4 hours a day		About 4 hours a day	
About 5 or more hours a day		About 5 or more hours a day	
6 or more hours a day		6 or more hours a day	

How many hours per day does your child usually spend watching television (including videos), at home or somewhere else, in his/her free time?

Weekdays		Weekends	
Never		Never	
Less than 1 hour per day		Less than 1 hour per day	
About 1 hour a day		About 1 hour a day	
About 2 hours a day		About 2 hours a day	
About 3 hours a day		About 3 hours a day	
About 4 hours a day		About 4 hours a day	
About 5 or more hours a day		About 5 or more hours a day	
